# Blue light transurethral resection and biopsy of bladder cancer with hexaminolevulinate: Histopathological characteristics and recurrence rates in a single UK centre study

**DOI:** 10.1002/bco2.250

**Published:** 2023-05-08

**Authors:** Kimberley Chan, Alexander Hampson, John Hayes, Joshua Rabinowitz, Nikhil Vasdev

**Affiliations:** ^1^ Lister Hospital East and North Hertfordshire NHS Trust Stevenage UK; ^2^ School of Life and Medical Sciences University of Hertfordshire Hatfield United Kingdom

**Keywords:** bladder tumour, blue light cystoscopy, carcinoma in situ, hexaminolevulinate, HexVix, TURBT

## Abstract

**Introduction:**

Blue light cystoscopy with hexaminolevulinate (HAL) during transurethral resection of bladder cancer (TURBT) has been shown to improve detection, thereby reducing bladder cancer recurrence compared with white light cystoscopy.

**Methods:**

Single‐centred UK (United Kingdom) study on 101 patients who underwent blue light cystoscopy between July 2017 and November 2020, performed by a single surgeon. Our study was divided into two arms; the primary arm had no prior diagnosis of bladder malignancy (*N* = 41), whereas secondary re‐resection arm had (*N* = 57). Three patients with non‐urothelial bladder cancer were excluded. Patients were followed up for 24 months. Data were collected on biopsy quality, histopathological characteristics and recurrence. The end points of the study were recurrence rate at 24 months in both arms and detection of CIS in patients who undergo TURBT or biopsy after initial white light study in the secondary, re‐resection arm. This was analysed with Fisher's exact test.

**Results:**

Of 98 patients, 39 had malignancy in their first blue light TURBT/biopsy: primary arm (10/41, 24.4%) and secondary arm (29/57, 50.9%), with detrusor present in 80.5% and 80.7%, respectively. In the secondary arm, blue light re‐resection TURBT detected significantly more CIS (20.7% vs 51.7%, *p* = 0.0277) compared with white light with 3.4% upstaged to muscle invasive bladder cancer (G3pT2). Median time to re‐resection was 3.06 months. Recurrence rate was 33.3% in the primary arm and 37.5% in the secondary arm after 24 months of follow‐up.

**Conclusion:**

Our data confirm that blue light TURBT with HAL provides superior detection and diagnosis of CIS in patients with previous white light cystoscopy.

## 1 INTRODUCTION

According to GLOBOCAN data, more than 570 000 cases of bladder cancers were diagnosed in 2020, making BC the 11th most common cancer worldwide. In the United Kingdom, bladder cancer is the ninth most common cancer, with more than 12 000 cases diagnosed annually.

The majority of BCs are urothelial cancer, making up 90% of all cases. Urothelial bladder cancer consists of non‐muscle invasive bladder cancer (NMIBC) and muscle invasive bladder cancer (MIBC). Approximately 75% of newly diagnosed bladder cancer are NMIBC at presentation,^1^ which histologically consist of tumours confined to mucosa [pTa, carcinoma in situ (CIS)] and lamina propria (pT1).

The gold standard treatment of NMIBC is transurethral resection of bladder tumour (TURBT) under white light cystoscopy (WLC). The main goals of TURBT are to completely resect any visible tumour to provide pathological material for accurate staging and to determine the presence, depth and type of tumour invasion.^2^ Rate of recurrence and progression after TURBT for NMIBC, however, could be as high as 60%–70% and 20%–30%, respectively, depending on the grade of bladder tumour.^3^ Failure to detect satellite lesions, small papillary tumours and CIS in WLC could lead to progression to invasive tumour, whereas incomplete resection and diffuse premalignant field result in recurrence. A study showed residual non‐invasive tumour in 75% of patients with superficial bladder tumour in their repeat TURBT within 2–6 weeks after initial resection, with 28% upstaged to invasive tumour.^4^

Furthermore, identification of CIS could be difficult in WLC. Novel therapies such as photodynamic diagnosis (PDD) have therefore been developed to enhance tumour detection and guide resection. PDD involves the instillation of photosensitiser hexaminolevulinate (HAL) into the urinary bladder via a urethral catheter 1 h before cystoscopy. HAL is a haem precursor of photoactive intermediate protoporphyrins IX (PpIX), which preferentially accumulates in neoplastic tissue due to increased mitotic rate. Exposure to blue light (wavelength 380–450 nm) results in activation of PpIX, emitting a pink‐red colour. Neoplastic tissue therefore appears pink‐red and demarcated, as opposed to normal bladder mucosa, which appears dark blue. This helps in the identification of bladder cancer. One meta‐analysis showed that blue light cystoscopy (BLC) detected significantly more CIS lesions than WLC. The same meta‐analysis also showed that in 26.7% of patients, CIS was only detected by blue light, which was significant in both patients with recurrent and primary bladder cancer.^5^

BLC was introduced in our trust in 2017. We report results from our site since its introduction. We assessed BLC with HAL (BLC‐HAL) on tumour recurrence rate at 24 months and evaluated detection of CIS in patients who undergo re‐resection BLC‐HAL‐TURBT/biopsy after initial white light TURBT/biopsy.

## 2 MATERIAL AND METHODS

### 2.1 Patients

101 patients with suspected bladder cancer based on outpatient cystoscopy underwent BLC‐HAL +/− TURBT +/− biopsy between July 2017 and November 2020.

Inclusion criteria for referral to BLC‐HAL in the primary arm (see Section 2.3) are multiple red patches suggestive of CIS on flexible cystoscopy (FC), atypical urine cytology, high‐grade disease suspected upon FC and patients with history of keratinising squamous metaplasia of bladder. In the secondary (re‐resection) arm, patients with first diagnosis of high‐grade disease or who have incomplete resection (where no muscle has been identified in histology) on initial white light TURBT/biopsy was included. In 2016, the World Health Organisation (WHO) classified bladder cancers based on differentiation as low grade or high grade.^6^ The distinction between low‐grade and high‐grade urothelial disease is important as it has implications related to risk stratification and management of patients.

### 2.2 HAL treatment and fluorescence detection

HexVix© was supplied as 85 mg of active ingredient HAL in 50 mL of phosphate saline diluent. Patients were asked to completely empty their bladder before HexVix© was instilled into the urinary bladder via a urethral catheter. This was retained in the bladder for 1 h and drained in the operating theatre immediately before cystoscopy. A D‐light system provided by Karl Storz was used for fluorescence detection, together with a PDD‐compatible telescope. A push button on the charge‐coupled camera head allows active operating modes of white and blue light to be used and switched between.

### 2.3 Study protocol

The study was divided into two arms. Patients in the primary arm had no prior diagnosis of bladder cancer and were referred for BLC biopsy/TURBT based on inclusion criteria. Patients in the secondary arm had been recently diagnosed with bladder cancer from initial white light TURBT/biopsy and were referred to re‐resection BLC TURBT/biopsy based on inclusion criteria. All BLC‐HAL biopsy/TURBT were performed by a single surgeon.

In the primary and secondary arms, patients with papillary or suspicious lesions seen in BLC‐HAL were excised and biopsied, respectively. The samples were analysed by local pathologists in our trust, who were not involved in the design of this study. The pathology report incorporated both the 1973 WHO and 2004/2016 WHO histological grading of bladder tumour.

Patients with confirmed malignancy in BLC were followed up with white light FC, with the interval for check cystoscopy dependent on the grade and stage of bladder cancer. Patients with suspected bladder cancer on check cystoscopy were re‐referred for white light biopsy +/− TURBT to check for recurrence.

### 2.4 Analysis

Pathological data were collected for patients with BLC malignancy confirmed by local pathologist in both arms. In the secondary arm, for patients with malignancy confirmed on BLC‐HAL biopsy/TURBT, retrospective data were collected on their initial pathology in white light. Based on the pathological data, patients were allocated to different prognostic risk groups based on 2021 European Association of Urology (EAU) scoring model.^7^ In cases of multifocal tumour +/− CIS, the highest grade and stage +/− CIS was recorded, with the presence of CIS primarily noted in such cases. The primary detection end point is the proportion of histologically confirmed malignancy in white light who had additional histologically confirmed CIS detected or upstaging in BLC re‐resection. Patients with non‐urothelial bladder cancer and patients with metastatic bladder cancer were excluded.

The primary and secondary arms were followed up for recurrence. Follow‐up was for 24 months from the date of first BLC TURBT/biopsy confirming malignancy. The primary recurrence endpoint is the proportion of patients with histologically confirmed bladder cancer (CIS, Ta, T1) in BLC TURBT/biopsy who were subsequently diagnosed with recurrent, histologically proven bladder tumour (CIS, Ta, T1–T4) within 24 months of follow‐up. Patients with non‐urothelial bladder cancer, a negative pathology in their first BLC, who passed away or had cystectomy after BLC were not followed up for recurrence.

The primary detection and recurrence endpoint was analysed by Fisher's exact test, performed by a single investigator. Median age (25th, 75th) was compared by Mann–Whitney *U* test, whereas gender and risk groups were analysed with chi‐squared test. Statistical significance was set at *p* < 0.05.

## 3 RESULTS

### 3.1 Cohort characteristics

Between July 2017 and November 2020, 101 patients underwent BLC TURBT or biopsy. 41 and 57 patients were enrolled to the primary and secondary arms, respectively. 3 patients were excluded from analysis as they had previous non‐urothelial bladder cancer. In the primary arm, BLC referral indications were 60.9% (25/41) for red patch suggestive of CIS in WLC, 17.1% (7/41) for atypical urine cytology, 12.2% (5/41) for high‐grade disease suggested on WLC, and 9.8% (4/41) for history of keratinising squamous metaplasia of bladder. In the secondary arm, 33.3% (19/57) were referred to BLC re‐resection for high‐grade disease and 66.7% (38/57) for incomplete resection in WLC. In both arms, 43.9% had BLC biopsy, whereas 56.1% had BLC TURBT (Table 1). In the primary and secondary arms, median age (25th, 75th) was 73.5 (65.0–76.8) and 73.0 (67–79.5), respectively (*p* = 0.6672). There were 87.8% (36/41) and 73.7% (42/57) male patients in the primary and secondary arms, respectively (*p* = 0.0871). Overall, 39 patients were found to have malignancy in their first BLC biopsy/TURBT, confirmed by histology: primary arm (10/41, 24.4%) and secondary arm (29/57, 50.9%). Risk groups for patients with malignancy confirmed on BLC biopsy/TURBT were 10.0% versus 6.9% low risk, 10.0% versus 27.6% intermediate risk, 40.0% versus 55.2% high risk and 40.0% versus 6.9% very high risk bladder cancer in the primary and secondary arms, respectively (Table 1). Regarding resection/biopsy quality, detrusor was present in 80.5% and 80.7% of samples in the primary and secondary arms, respectively.

**TABLE 1:** Clinical and pathological characteristics of patients enrolled to blue light cystoscopy in both primary and secondary arms.

	Primary arm	Secondary arm
Cohort characteristics and study arm		
Patients enrolled	41	57
Median age (25, 75)	73.5 (65.0–76.8)	73 (67.0–79.5)
Male	36 (87.8)	42 (73.7)
BLC‐HAL biopsy	18 (43.9)	25 (43.9)
BLC‐HAL TURBT	23 (56.1)	32 (56.1)
NMIBC at BLC‐HAL TURBT/biopsy	10 (24.4)	29 (50.9)
NMIBC at BLC‐HAL TURBT	8 (80.0)	17 (58.6)
NMIBC at BLC‐HAL biopsy	2 (20.0)	12 (41.4)
Indication categories		
Red patch suggestive of CIS in WLC	25 (60.9%)	—
Atypical urine cytology	7 (17.1%)	—
High‐grade disease suggested on WLC	5 (12.2%)	—
History of keratinising squamous metaplasia of bladder	4 (9.8%)	—
Re‐resection for high‐grade disease in WLC	—	19 (33.3%)
Re‐resection for incomplete resection in WLC	—	38 (66.7%)
Risk groups for patients with blue light malignancy		
Low	1 (10.0%)	2 (6.9%)
Intermediate	1 (10.0%)	8 (27.6%)
High	4 (40.0%)	16 (55.2%)
Very high	4 (40.0%)	2 (6.9%)
MIBC	—	1 (3.4%)

*Note*: All patients (*N* = 98), three patients with non‐urothelial bladder cancer excluded.

### 3.2 Detection

**FIGURE 1:**
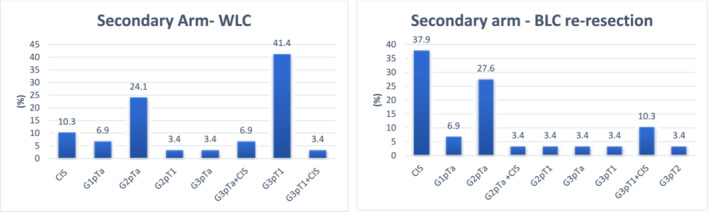
Graphs showing pathology of patients in secondary arm who had WLC (left) and subsequent BLC re‐resection (right).

The primary detection endpoint was the proportion of histologically confirmed malignancy in white light who had additional histologically confirmed CIS detected or upstaging in BLC re‐resection. Of 29 patients who had BLC re‐resection confirming malignancy, 62.1% (18/29) had TURBT, and the remaining had biopsy. A statistically significant proportion of patients had CIS detected in re‐resection BLC TURBT/biopsy compared with respective white light studies (*p* = 0.0277). In the secondary arm, 20.7% (6/29) patients had CIS on initial WLC‐TURBT/biopsy. BLC re‐resection detected CIS in 51.7% (15/29) of patients (Table 2). 31.0% (9/29) had additional CIS detected on BLC alone. Furthermore, 3.4% (1/29) were upstaged to MIBC (Figure 1), although this was not statistically significant (*p* = 1.0). After BLC‐HAL re‐resection, 20.7% (6/29) patients had a higher 2021 EAU NMIC risk group scoring. In particular, three patients with high‐risk bladder cancer were classified to have very‐high‐risk bladder cancer after BLC re‐resection showed additional inclusion of CIS. Overall, 55.2% (16/29) had re‐resection within 3 months, with median time to re‐resection being 3.06 months.

**TABLE 2:** Pathological data on patient who had WLC and subsequent malignancy on BLC re‐resection.

Patient #	WLC grade	Risk group	BLC re‐resection grade	Risk group
1	G3pT1	High	G3pT1	High
2	G2pTa	Intermediate	G3pTa	High
3	G1pTa	Low	G1pTa	Low
4	G3pT1 + CIS	Very high	CIS	High
5	G1pTa	Low	CIS	High
6	G3pT1	High	CIS	High
7	G2pT1	High	G2(HG)pTa	Intermediate
8	G3pT1	High	CIS	High
9	G3pT1	High	G2(LG)pTa + CIS	High
10	G2(LG)pTa	Intermediate	G2pTa	Intermediate
11	CIS	High	CIS	High
12	CIS	High	CIS	High
13	G2(HG)pTa	Intermediate	G1pTa	Low
14	G3pT1	High	G2pTa	Intermediate
15	G3pT1	High	CIS	High
16	G3pT1	High	G3pT1 + CIS	Very high
17	G3pTa	High	G2pTa	Intermediate
18	G3pT1	High	G3pT1 + CIS	Very high
19	G2(LG)pTa	Intermediate	G2(LG)pTa	Intermediate
20	G2(HG)pTa	Intermediate	G2pT1	High
**21**	**G3pT1**	**High**	**G3pT2**	**MIBC**
22	CIS	High	CIS	High
23	G2(LG)pTa	Intermediate	G2pTa	Intermediate
24	G3pTa + CIS	High	CIS	High
25	G2pTa	Intermediate	G2(LG)pTa	Intermediate
26	G3pT1	High	G3pT1 + CIS	Very high
27	G3pT1	High	CIS	High
28	G3pTa + CIS	High	CIS	High
29	G3pT1	High	G2pTa	Intermediate

*Note*: Patient # coloured in grey indicate increase in risk group after BLC re‐resection; patient # bolded indicate upstaging to MIBC.

Abbreviations: HG, high grade; LG, low grade.

### 3.3 Recurrence

**FIGURE 2:**
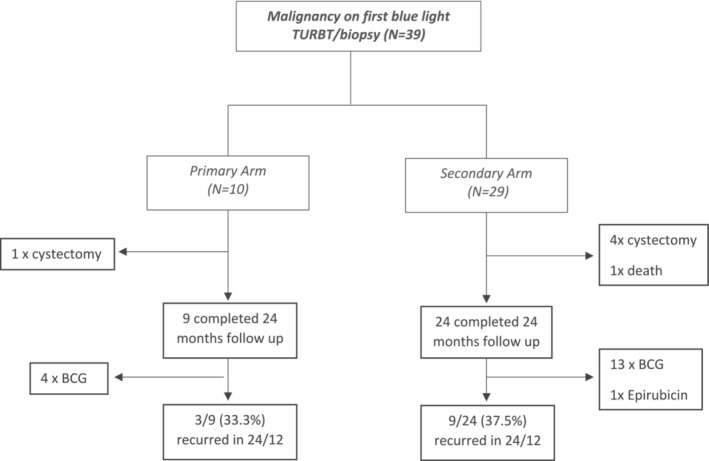
Flowchart of patients in primary and secondary arms being followed up for 24 months.

All patients were followed up for 24 months after diagnosis of blue light malignancy (Figure 2). 6 patients were excluded from follow‐up: 5 patients had cystectomy after diagnosis of blue light malignancy, whereas 1 died during the follow‐up period. Overall, recurrence rate at 24 months was 33.3% (3/9) in the primary arm and 37.5% (9/24) in the secondary arm. In the primary arm, among the 3 patients who recurred, very high risk: high risk bladder cancer = 1:2, 2 patients received Bacillus Calmette–Guérin (BCG) after BLC resection. In the secondary arm, among the 9 patients who recurred, high risk: intermediate risk: low risk bladder cancer = 6:2:1, 6 patients received BCG treatment post BLC re‐resection, and 50% who received BCG treatment had high risk bladder cancer.

## 4 DISCUSSION

Results from our single UK centre confirms that BLC with intravesical HAL improves detection of CIS with statistical significance. Our findings support multiple studies published in the literature. A prospective randomised trial in 2013 of BLC with HAL versus white light showed that intravesical HAL was an effective diagnostic tool for CIS. Secondary CIS was identified in 26% of HAL‐PDD arm compared with 14% in the white light arm (*p* = 0.04).^8^ These figures correlate with our results, with 51.7% CIS detected in the BLC re‐resection arm as opposed to WLC‐TURBT (*p* = 0.0277). Apart from improved detection of CIS, addition of BLC with TURBT has been shown to prolong time to progression from Ta to CIS relative to WLC. This was reported in a study that randomised patients to undergo either WL cystoscopy followed by TURBT versus additional inspection with BLC before TURBT, followed up for a median of 55.1 and 53.0 months, respectively (*p* = 0.05).^9^

Given the encouraging results of BLC on CIS detection in our study, it is unsurprising that some centres have already incorporated office‐based blue light flexible cystoscopy (BLFC) in their practice. This makes sense if BLFC has the potential advantage of finding cancer in normal WLC. A recent prospective study on patients with NMIBC who underwent office‐based BLFC yield positive results. Office‐based BLFC detected 33% additional cancer in patients with positive WLC and BLFC.^10^ This could be an effective way of providing bladder cancer surveillance in high‐risk patients, but further research needs to be done on its cost‐effectiveness.

Another important finding in our present study is 3.4% patients being upstaged to MIBC on blue light re‐resection. This patient subsequently underwent robotic‐assisted laparoscopic cystoprostatectomy, pelvic lymph node dissection and extracorporeal ilea conduit formation, after upstaging from G3pT1 to G3pT2. Although no statistical significance can be concluded from this, BLC's supportive role in surgical management cannot be overlooked. A study on patients with clinical T2 bladder cancer showed that a cystectomy delay of 3.1 months undermined patient survival and is associated with a worse oncological outcome.^11^

Multiple systematic reviews have reported encouraging results on blue light TURBT in reducing recurrence rates compared with white light studies.^12^, ^13^ One systematic review concluded that blue light TURBT reduces disease recurrence in higher NMIBC risk groups than lower NMIBC risk groups. They report 147 fewer recurrences per 1000 in patients with high‐risk NMIBC versus 48 in patients with low‐risk NMIBC after blue light TURBT. A recently published ‘PHOTO’ trial randomised patients with suspected first diagnosis of bladder cancer with intermediate or high risk of recurrence based on routine visual assessment to either PDD‐TURBT or WLC‐TURBT. This study reported no benefit of PDD‐TURBT on reducing recurrence rates at 3 years.^14^ However, there were several shortcomings in the study. This was nicely analysed by Stenzl et al.'s editorial.^15^ It highlighted that the trial was underpowered and skewed by selection bias, with 20% patients excluded after randomisation. These factors need to be taken into account before overriding previous published studies. We report a recurrence rate of 33.3% and 37.5% in the primary and secondary arms after 24 months of follow‐up. The absence of a matched cohort of WLC patients limits our interpretation as to whether BLC reduces recurrence rate compared with WLC. We could however comment that, with the majority of patients in the primary and secondary arms belonging to high‐ and very‐high‐risk groups in both arms (80% in primary and 62.1% in secondary arm), the incidence of recurrence is relatively low compared to published data on recurrence rate for patients with high‐grade NMIBC. A population‐based analysis on patients with high‐risk NMIBC reported a recurrence rate of 61.1% at 2 years.^16^

Our report has limitations. Firstly, our patients were not randomised to undergo BLC in the primary and secondary group. We were not able to randomise patients for BLC‐HAL as this was a new technique introduced in our trust back in 2017, with only one surgeon performing BLC at limited capacity. Hence, patients were referred based on inclusion criteria. Secondly, a surprisingly large number of patients referred for BLC TURBT/biopsy did not have malignancy. 60.2% patients did not have malignancy on histology. Among such, indications of referral to BLC for many patients were ‘red patches suggestive of CIS’, but final pathology only showed chronic inflammation or cystitis. In our current practice, a large proportion of diagnostic cystoscopies are performed by junior doctors of the urology team. Patients were referred for BLC based on their clinical suspicion of CIS. Their diagnostic skills might not match urology consultants. This might explain the low yield of cancer cases in our study. Finally, our sample size for BLC is small. With BLC being a relatively new technique introduced in our trust in 2017, performed by a single surgeon only, coupled with the effects of the COVID‐19 pandemic made high‐volume BLC‐HAL difficult during 2017–2020.

## 5 CONCLUSION

We report our experience as a single UK centre introducing BLC in our diagnostics. We report superior detection of CIS in patients who had BLC re‐resection with statistical significance. We aim to continue recruiting patients with suspected CIS for BLC to improve detection and thereby guide management.

## AUTHOR CONTRIBUTIONS

Kimberley Chan contributed to the design and implementation of the research, to the analysis of the results and to the writing of the manuscript. Alexander Hampson contributed to data collection. John Hayes contributed to data collection. Joshua Rabinowitz contributed to data collection. Nikhil Vasdev contributed to design, implementation of research and supervision of work.

## CONFLICT OF INTEREST STATEMENT

I have no disclosures. I have also confirmed with all my co‐authors, who have no disclosures or conflict of interests.
